# Supplementary material for: Damage to the right insula disrupts the perception of affective touch
Source: eLife. 2020 Jan 24;9:e47895. doi: 10.7554/eLife.47895 (PMC7043887; doi:10.7554/eLife.47895)
Supplement: Supplementary file 1. — As control for a general pleasantness deficit, patients rated how pleasant it would be to be touched by a typically pleasant material (i.e. velvet, Mpleasantness rating = 6.91, SD = 1.88) and a typically unpleasant fabric (i.e. sandpaper, Mpleasantness rating = 0.33, SD = 0.93). Similarly, as for CT pleasantness sensitivity, top-down tactile pleasantness sensitivity was computed as the difference between pleasant (velvet) and unpleasant pleasantness ratings (sandpaper), for each patient. We considered the same patients as for the CT pleasantness sensitivity VLSM analysis (N = 36 as we had missing data for 5 of them) and ran a VLSM analysis with this top-down tactile pleasantness sensitivity. [file elife-47895-supp1.docx]

**Supplementary File 1. Number of significant voxels (from the atlas of grey matter – AAL – and white matter – JHU – and NatBrainLab’s atlas) resulting from the VLSM analysis with the general pleasantness sensitivity scores** (velvet-sandpaper average pleasantness ratings), N=36**.**

As control for a general pleasantness deficit, patients rated how pleasant it would be to be touched by a typically pleasant material (i.e. velvet, M_pleasantness rating_ = 6.91, SD = 1.88) and a typically unpleasant fabric (i.e. sandpaper, M_pleasantness rating_ = 0.33, SD = 0.93). Similarly, as for CT pleasantness sensitivity, top-down tactile pleasantness sensitivity was computed as the difference between pleasant (velvet) and unpleasant pleasantness ratings (sandpaper), for each patient. We considered the same patients as for the CT pleasantness sensitivity VLSM analysis (N=36 as we had missing data for 5 of them) and ran a VLSM analysis with this top-down tactile pleasantness sensitivity.

| **Regions necessary for imagined tactile pleasantness sensitivity** | | | | | | |
| --- | --- | --- | --- | --- | --- | --- |
| **AAL** | **Region** | **Volume** | **x** | **y** | **z** | **T-value** |
|  | Unclassified | 111 | 24 | -18 | 6 | 3.23 |
|  | Caudate | 48 | 13 | 14 | 4 | 2.85 |
|  | Putamen | 14 | 21 | 4 | 8 | 2.69 |
|  | Pallidum | 69 | 22 | -5 | 7 | 2.86 |
|  | Thalamus | 19 | 20 | -13 | 7 | 2.96 |
| **JHU** | Unclassified | 22 | -5 | 7 | 22 | 2.86 |
|  | Anterior_limb_of_int | 15 | 14 | 4 | 15 | 2.85 |
|  | Posterior_limb_of_in | 24 | -18 | 6 | 24 | 3.23 |
|  | Retrolenticular_part | 25 | -22 | 7 | 25 | 2.60 |
|  | Posterior_corona_rad | 27 | -34 | 25 | 27 | 2.53 |
| **NatBrainLab** | Unclassified | 82 | 13 | 14 | 4 | 2.85 |
|  | Corpus_Callosum | 1 | 22 | -28 | 28 | 2.53 |
|  | Cortico_Ponto_Cerebellum | 6 | 19 | -10 | 12 | 2.49 |
|  | Cortico_Spinal | 96 | 23 | -18 | 6 | 3.10 |
|  | Internal_Capsule | 76 | 24 | -18 | 6 | 3.23 |
