# Supplementary material for: Damage to the right insula disrupts the perception of affective touch
Source: eLife. 2020 Jan 24;9:e47895. doi: 10.7554/eLife.47895 (PMC7043887; doi:10.7554/eLife.47895)
Supplement: Transparent reporting form [file elife-47895-transrepform.pdf]

eLIFE
1st Floor
24 Hills Road
Cambridge CB2 1JP, UK
P 01223 855340
W elifesciences.org
T @elife

eLife's transparent reporting form

We encourage authors to provide detailed information *within their submission* to facilitate the interpretation and replication of experiments. Authors can upload supporting documentation to indicate the use of appropriate reporting guidelines for health-related research (see EQUATOR Network), life science research (see the BioSharing Information Resource), or the ARRIVE guidelines for reporting work involving animal research. Where applicable, authors should refer to any relevant reporting standards documents in this form.

If you have any questions, please consult our Journal Policies and/or contact us: editorial@elifesciences.org.

Sample-size estimation

- You should state whether an appropriate sample size was computed when the study was being designed
- You should state the statistical method of sample size computation and any required assumptions
- If no explicit power analysis was used, you should describe how you decided what sample (replicate) size (number) to use

Please outline where this information can be found within the submission (e.g., sections or figure legends), or explain why this information doesn't apply to your submission:

The sampling procedure was random, and dependent upon stroke wards referrals. We used statistical software GPower (version 3.1.2, http://www.psycho.uni-duesseldorf.de/abteilungen/aap/gpower3/) to calculate a priori the required sample. With parameters set to: alpha=.05, measurements=2 (CT-optimal vs. CT-suboptimal velocities), Group=2 (Stroke patients vs. Healthy controls), nonsphericity correction=1 and correlation among repeated measures = 0.5, we would have sufficient power = .80 to detect a small/medium effect size f=.30 with a sample size N=20. Given this calculation, we decided to test 20 healthy controls and to have a minimum of 20 patients that met our criteria in all conditions, which led us to test 59 patients.

Replicates

- You should report how often each experiment was performed
- You should include a definition of biological versus technical replication
- The data obtained should be provided and sufficient information should be provided to indicate the number of independent biological and/or technical replicates
- If you encountered any outliers, you should describe how these were handled
- Criteria for exclusion/inclusion of data should be clearly stated
- High-throughput sequence data should be uploaded before submission, with a private link for reviewers provided (these are available from both GEO and ArrayExpress)

Please outline where this information can be found within the submission (e.g., sections or figure legends), or explain why this information doesn't apply to your submission:

eLife Sciences Publications, Ltd is a limited liability non-profit non-stock corporation incorporated in the State of Delaware, USA, with company number 5030732, and is registered in the UK with company number FC030576 and branch number BR015634 at the address 1st Floor, 24 Hills Road, Cambridge CB2 1JP | August 2014

We report here only one experiment in a relatively large sample of stroke patients, but with no replication.
All behavioural and neuropsychological data is accessible in open access on Open Science Framework (https://osf.io/fyrwc/?view_only=75773c749be84432994beca994481988).

Statistical reporting

- Statistical analysis methods should be described and justified
- Raw data should be presented in figures whenever informative to do so (typically when N per group is less than 10)
- For each experiment, you should identify the statistical tests used, exact values of N, definitions of center, methods of multiple test correction, and dispersion and precision measures (e.g., mean, median, SD, SEM, confidence intervals; and, for the major substantive results, a measure of effect size (e.g., Pearson's r, Cohen's d)
- Report exact p-values wherever possible alongside the summary statistics and 95% confidence intervals. These should be reported for all key questions and not only when the p-value is less than 0.05.

Please outline where this information can be found within the submission (e.g., sections or figure legends), or explain why this information doesn't apply to your submission:

See Material and Methods Section 4 (behavioural) & 5 (lesion mapping)

Behavioral data was analyzed with SPSS version 23 (IBM, Armonk, NY).
Lesion drawing, overlays, white and grey matter identification were carried out with MRIcron software (http://www.cabiatl.com/mricro/mricron/index.html).
Voxel-based lesion symptom mapping was conducted via Matlab 2015a (Mathworks, 2002), and Matlab code from Bates et al. (2003, available on https://aphasialab.org/vlsm/)

(For large datasets, or papers with a very large number of statistical tests, you may upload a single table file with tests, Ns, etc., with reference to sections in the manuscript.)

Group allocation

- Indicate how samples were allocated into experimental groups (in the case of clinical studies, please specify allocation to treatment method); if randomization was used, please also state if restricted randomization was applied
- Indicate if masking was used during group allocation, data collection and/or data analysis

Please outline where this information can be found within the submission (e.g., sections or figure legends), or explain why this information doesn't apply to your submission:

See Material and Methods section 1.

Fifty-nine, unilateral, right-hemisphere-lesioned stroke patients (mean age: 65.86 ± 14.12 years; age range: 38-88 years; 31 females) were recruited from consecutive admissions to seven stroke wards as part of a larger study; using the following inclusion criteria: (i) imaging- confirmed first ever right hemisphere lesion; (ii) contralateral hemiplegia; (iii) <4 months from symptom onset; (iv) no previous history of neurological or psychiatric illness; (v) >7 years of education; (vi) no medication with significant cognitive or mood side-effects; (vii) no language impairments that precluded completion of the study assessments; and (viii) right handed.

Twenty age-matched healthy control subjects were recruited and tested with the same behavioral paradigm in order to assess the specificity of deficits in the patient group (healthy control group; 63.05 ± 12.12 years; age range: 46-87 years; 11 females).

Stroke patients were referred by local nurses when they met the inclusion criteria. No bias in recruitment was therefore possible.
Control participants were recruited via the UCL Sona System.

Additional data files ("source data")

- We encourage you to upload relevant additional data files, such as numerical data that are represented as a graph in a figure, or as a summary table
- Where provided, these should be in the most useful format, and they can be uploaded as "Source data" files linked to a main figure or table
- Include model definition files including the full list of parameters used
- Include code used for data analysis (e.g., R, MatLab)
- Avoid stating that data files are "available upon request"

Please indicate the figures or tables for which source data files have been provided:

All behavioural and neuropsychological data is accessible in open access on Open Science Framework (https://osf.io/fyrwc/?view_only=75773c749be84432994beca994481988).
Lesion data can be available upon request.
